# Inversion for Refractivity Parameters Using a Dynamic Adaptive Cuckoo Search with Crossover Operator Algorithm

**DOI:** 10.1155/2016/3208724

**Published:** 2016-04-26

**Authors:** Zhihua Zhang, Zheng Sheng, Hanqing Shi, Zhiqiang Fan

**Affiliations:** Department of Space and Remote Sensing, College of Meteorology and Oceanography, PLA University of Science and Technology, Nanjing 211101, China

## Abstract

Using the RFC technique to estimate refractivity parameters is a complex nonlinear optimization problem. In this paper, an improved cuckoo search (CS) algorithm is proposed to deal with this problem. To enhance the performance of the CS algorithm, a parameter dynamic adaptive operation and crossover operation were integrated into the standard CS (DACS-CO). Rechenberg's 1/5 criteria combined with learning factor were used to control the parameter dynamic adaptive adjusting process. The crossover operation of genetic algorithm was utilized to guarantee the population diversity. The new hybrid algorithm has better local search ability and contributes to superior performance. To verify the ability of the DACS-CO algorithm to estimate atmospheric refractivity parameters, the simulation data and real radar clutter data are both implemented. The numerical experiments demonstrate that the DACS-CO algorithm can provide an effective method for near-real-time estimation of the atmospheric refractivity profile from radar clutter.

## 1. Introduction

Atmospheric duct can change the electromagnetic wave propagation path and effective coverage areas, as it is formed in an anomalous atmospheric refractivity structure. It can trap the wave within a certain atmosphere layer and form the atmospheric duct propagation [1]. In general, the low altitude propagation loss will be much less than for a standard atmosphere condition when a duct is existent [2]. Ducts can substantially influence the capability of various radio systems, such as radar, communication, reconnaissance, and some other electromagnetic radiation systems [3]. Therefore, forecasting the real-time atmospheric refractivity structure and analysing its influence on the electric systems have a great research value. Traditionally, atmospheric ducts can be measured via radiosondes or rocketsondes or using numerical weather prediction models [4]. However, these methods are too expensive and unable to provide real-time duct information [5]. In the previous work, it has been found that the temporal and spatial variations of radar clutter are related to the temporal and the spatial variations of the refractivity profile [6], which contributes to the research of atmospheric refractivity estimation based on RFC (Refractivity from Clutter). Using the RFC technique to estimate refractivity has many advantages, such as low costs, operation convenience, and high temporal and spatial resolutions of refractivity profiles [2]. However, it is very difficult to get the analytical solutions using RFC, because the relationship between refractivity parameters and radar clutter is clearly nonlinear and ill-posed. To deal with this problem, several inversion algorithms have been used in RFC. Gerstoft et al. proposed using the genetic algorithm (GA) to perform global refractivity estimation [1]. Yardim et al. used the hybrid algorithm GA-MCMC and the Kalman and particle filters methods to research the RFC problem [2, 7]. Wang et al. used the particle swarm optimization (PSO) to estimate the evaporation duct heights [8]. Zhang et al. used the improved hybrid algorithm, particle swarm optimization via Lévy flight (LPSO), to estimate the five parameters of hybrid duct and analysed its antinoise ability [9]. Sheng et al. applied a series of methods to research RFC [10–12]. However, in order to obtain a more precise solution, it is still required to find a more efficient and stable inversion algorithm.

Cuckoo search (CS) algorithm is a novel metaheuristic algorithm. The characteristics of CS are ease of implementation, presence of a few parameters to adjust, and being able to guarantee global convergence [13]. Cuckoo search algorithm has shown good performance both on benchmark unconstrained functions and on real-world problems, which has been applied in several fields [14–16]. However, the parameters of CS are kept constant and the algorithm lacks the mutation mechanism, which may lead to a poor convergence rate and easy trapping into local optimum in its late period. In order to improve the ability of the CS algorithm, a dynamic adaptive operation and a crossover operation (CO) are merged into the standard cuckoo search (DACS-CO). Rechenberg's 1/5 criteria combined with learning factor are used to control parameter dynamic adaptive adjusting process and the crossover operation is utilized to guarantee the population diversity. Using the RFC technique to estimate atmospheric refractivity is a complex nonlinear and ill-posed optimization problem. The DACS-CO algorithm has better local search ability and contributes to superior performance. Thus, in this paper, the new improved algorithm, DACS-CO, was selected as the optimization algorithm being utilized in the RFC technique to estimate the atmospheric refractivity. In order to illustrate the performance of DACS-CO, the simulation and the real data experiment are both implemented, and the retrieval results are compared with the CS, genetic algorithm (GA), and particle swarm optimization (PSO) algorithms.

The rest of this paper is organized as follows. The basic theory and parameters model are introduced in Section 2. The DACS-CO algorithm is described in Section 3. The simulation experiment is in Section 4, and the layout and simulation results are also given. In Section 5, the real data experiment is presented. Finally, the conclusions are summarized in Section 6.

## 2. Theory and Model

### 2.1. Atmospheric Refractivity Model

**Figure 1:**
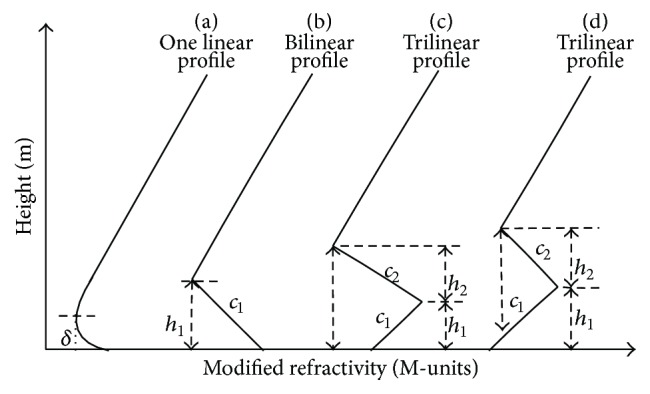
The four most typical duct types. (a) The evaporation duct. (b) The surface duct. (c) The surface-based duct. (d) The surface-based duct.

Atmospheric structures can be characterized by their vertical refractive index profile. To perform RFC, some idealized parameters estimation models were presented, such as one linear model, bilinear model, and trilinear model, which can be seen in Figure 1. The surface-based duct (Figure 1(c)) and elevated duct (Figure 1(d)) structures can be depicted by a four-parameter trilinear refractivity profile [7], which is represented by vector *m* = (*c*_1_, *c*_2_, *h*_1_, *h*_2_), where *m* is the modified index of atmospheric refraction. The value of *m* as a function of height *z* is given by

(1) Mz=M0+c1z0≤z≤h1,M0+c1h1+c2z−h1h1<z<h1+h2,M0+c1h1+c2h2+0.118z−h1−h2z>h1+h2,

where *M*_0_ is the modified index of refraction at the sea surface, usually taken as 330 M-units, *c*_1_ and *h*_1_ represent the slope and thickness of the base layers, and *c*_2_ and *h*_2_ represent the slope and thickness of the inversion layers. When *h*_1_ reduces to zero, the trilinear profile will end up with a bilinear profile, which means that the bottom of the duct touches the ground (Figure 1(b)). The trilinear model can properly estimate the refractivity M-profile via RFC method. Thus, we select the trilinear atmospheric refractivity profile model to research in this study.

### 2.2. Radar Clutter Power

The clutter signal power *P*_*c*_ received by the radar can be calculated by [17]

(2) $$\begin{array}{c} P_{c}=\frac{4\pi P_{t}G_{t}G_{r}A_{c}\sigma^{0}}{L_{\text{loss}}^{2}\lambda^{2}}, \end{array}$$

where *P*_*t*_ is the transmitted power, *G*_*t*_ is the transmitted antenna gain, *G*_*r*_ is the receiving antenna gain, *λ* is the wavelength, *A*_*c*_ is the illuminated area, and *σ* ^0^ is the normalized radar cross section of sea clutter. *L*_loss_ is the one-way propagation loss, which is given by

(3) $$\begin{array}{c} L_{\text{loss}}=20{\;\log}_{10}\left(\;\frac{4\pi x}{\lambda }\;\right)-20{\;\log}_{10}F; \end{array}$$

here, *F* is the propagation factor, which is expressed as follows:

(4) $$\begin{array}{c} F=\sqrt{x}\left|\;u\left(\;x,z\;\right)\;\right|. \end{array}$$

At low grazing angles, *A*_*c*_ is a linear function of range *r*. Letting the symbols *P*_*c*_, *σ* ^0^, and *L*_loss_ be all in units of dB, the clutter signal power from the clutter can be modelled as [1]

(5) $$\begin{array}{c} P_{c}=-2L_{\text{loss}}+10{\;\log}_{10}\left(\;r\;\right)+\sigma^{0}+C, \end{array}$$

where *σ* ^0^ is the radar cross section of the sea surface and *C* is a constant that includes radar parameters, such as the transmitted power and antenna pattern.

In order to convert the parameter estimation problem into optimization problem, a simple least squares objective function can be defined as

(6) $$\begin{array}{c} f\left(\;m\;\right)=e^{T}e, \end{array}$$

where

(7) $$\begin{array}{c} e=P_{c}^{\text{obs}}-P_{c}\left(\;m\;\right). \end{array}$$

Here, *P*_*c*_^obs^ is the observed radar clutter power and *P*_*c*_ is the modeled radar clutter power. *m* = (*c*_1_, *c*_2_, *h*_1_, *h*_2_) is the modified index of atmospheric refraction, which is a function of height *z* given as (1).

### 2.3. The Terrain Parabolic Equation Model

In spherical coordinate system, the vector wave equations could be transformed into Helmholtz equation. Combined with Earth-Flattening transform, conformal mapping, and scale analysis, Helmholtz equation could be converted into TPE (terrain parabolic equation) [18]. Tropospheric radiowave propagation over the sea is presented commonly as the terrain parabolic equation model.

Because of the temporal and spatial inhomogeneity of *n*, it is very difficult to accurately figure out TPE. At present, the Fourier split-step algorithm is prevalently accepted. For range *x* and height *z*, if *u*(*x*, *z*) is the electromagnetic field, then the field in range *x* + Δ*x* and at height *z*, denoted by *u*(*x* + Δ*x*, *z*), can be calculated by the split-step Fourier solution [19], which is defined by

(8) ux+Δx,z=exp⁡ikΔxm2−12×F−1exp⁡−iP2δr2kFux,z,

where *m* is the modified index of refraction, *F* and *F* ^−1^ are, respectively, the Fourier transform and inverse Fourier transform, *P* = *k*sin⁡*η* is the vertical wave number or the spatial frequency, and *η* is the propagation angle from the horizontal direction. *z* and *P* are associated by *zP* = *Nπ*, and *N* is the discrete Fourier transform size.

## 3. DACS-CO Algorithm

DACS-CO is a new hybrid algorithm, the core thought of which is integrating the parameter dynamic adaptive adjusting process and crossover operation into the standard cuckoo search (CS) algorithm. Rechenberg's 1/5 criteria were used to control parameters of the algorithm dynamic adjusted. The crossover operation is utilized to guarantee the population diversity.

### 3.1. Cuckoo Breeding Behavior and Lévy Flights

Cuckoo search algorithm is a nature inspired metaheuristic algorithm proposed by Yang and Deb [20]. They combined the cuckoo breeding behavior with Lévy flights. Cuckoo algorithms attract the attention of the scientists all over the world because of their fascinating breeding behavior such as the aggressive reproduction strategy. Some cuckoos reproduce via laying their eggs in nests of other host birds, removing the other bird eggs to increase their reproductivity [21]. It is worth mentioning that cuckoo eggs may be discovered by the host birds. In this case, host birds will either take off the alien eggs or simply abandon their nests and build new ones elsewhere. In the process of evolution, some female parasitic cuckoos can imitate the colors and patterns of the eggs of a few chosen host species [20]. This reduces the probability of the eggs being abandoned and thus increases their incubation.

In order to simplify describing the cuckoo search, Yang and Deb [20] used the following three idealized rules:

- Each cuckoo lays one egg at a time and dumps it in a randomly chosen nest.
- The best nests with high quality of eggs (solutions) will carry over to the next generations.
- The number of available host nests is fixed, and a host can discover an alien egg with a probability. In this case, the host bird can either throw the egg away or abandon the nest so as to build a completely new nest in a new location.

The term “Lévy flight” was coined by Mandelbrot [22]; many studies have shown that the flight behavior of real birds, insects, grazing animals, and fish has demonstrated the typical feature of Lévy flights [23]. Reynolds and Frye [24] showed that fruit flies or* Drosophila melanogaster* explore their landscape using a series of straight flight paths punctuated by a sudden 90-degree shift, leading to a Lévy-flight-style intermittent scale free search pattern. The large steps occasionally taken make the algorithm suitable for global search. Such behavior has been applied to cuckoo search algorithm; the large steps occasionally taken make the algorithm suitable for global search [9].

Each cuckoo egg can be regarded as a solution. Let *X* = (*x*_1_, *x*_2_,…,*x*_*k*_)^*T*^ ∈ *R* ^*k*^ denote a solution. In the initial searching process, each solution is generated randomly. A solution *x*_*i*_ is updated to a new value with the use of Lévy flight, which is performed as per Yang and Deb [20]:

(9) $$\begin{array}{c} X_{i}^{\left(t+1\right)}=X_{i}^{\left(t\right)}+\alpha ⊕\text{Lévy}\left(\;\lambda \;\right), \end{array}$$

where

(10) $$\begin{array}{c} \text{Lévy}\left(\;\lambda \;\right)=\frac{\lambda \Gamma \left(\lambda \right)\sin\left(\pi \lambda /2\right)}{\pi s^{1+\lambda }}; \end{array}$$

here, *α* > 0 is the step size. The product ⊕ means entrywise multiplications, and Lévy(*λ*) is a Lévy flight in which the step lengths are distributed according to the following probability distribution:

(11) $$\begin{array}{c} \text{Lévy}\left(\;\lambda \;\right)\sim u=t^{-\lambda },\;\left(1<\lambda \leq 3\right). \end{array}$$

### 3.2. Parameters Adjustment by Rechenberg's 1/5 Criteria

The performance of CS algorithm greatly depends on the parameters *p*_*α*_ and *α*, where *p*_*α*_ is the probability of abandoning the worse nests and *α* is the step size. The standard CS algorithm uses fixed values for both *p*_*α*_ and *α*. The parameter values are determined before operation and cannot be changed during new generations. However, it is found that if the value of *p*_*α*_ is large and the value of *α* is small, the speed of convergence is high but the quality of solution is decreased; if the value of *p*_*α*_ is small and the value of *α* is large, the quality of solution is high but the performance will be poor and the number of iterations will increase a lot [25]. So it is difficult to determine a proper set of parameter values in the initialization step.

To improve the diversification and intensification of the population, a dynamic adaptive operation of algorithm parameters is integrated into CS. Rechenberg's 1/5 criteria combined with learning factor are used to evaluate evolution process [26]. Rechenberg proposed the principle of 1/5 on the study of the evolutionary computation, the principle that “the success of its variation ratio should be kept in the 1/5.” That is to say, the control parameters of the algorithm should be dynamically adjusted with the proportion of new solution success, and the ration should be maintained at 1/5. However, in the actual search, it is not often seen that the improved ratio is just as 0.2. In order to keep the parameters adjusting stably, the parameter range extends from just 0.2 to [0.2, 0.3] based on the original 1/5 principle. The detailed procedures for step size factor *α* are described as

(12) $$\begin{array}{c} \alpha^{t+1}=\left\{\begin{array}{cc} \frac{\alpha^{t}}{l_{\alpha }} & R<0.2,\\ \alpha^{t} & 0.2\leq R\leq 0.3,\\ \alpha^{t}*l_{\alpha } & R>0.3, \end{array}\right. \end{array}$$

where *l*_*α*_ is the learning factor of the step size. *R* is the improved ratio. Similarly, the abandoning probability factor *p*_*α*_ can be adjusted as

(13) $$\begin{array}{c} p_{\alpha }^{t+1}=\left\{\begin{array}{cc} \frac{p_{\alpha }^{t}}{l_{p_{\alpha }}} & R<0.2\\ p_{\alpha }^{t} & 0.2\leq R\leq 0.3\\ p_{\alpha }^{t}*l_{p_{\alpha }} & R>0.3. \end{array}\right. \end{array}$$

### 3.3. Crossover Operation

Cuckoo search algorithm is easily trapped into local optimum in its late period due to lack of population diversity. The crossover operation (CO) is integrated into the CS algorithm to add the information exchange between individuals. Generally, the crossover operation is used in genetic algorithm (GA) [27]. The procedure can be described as the following steps. (1) The individuals in the population are sorted by the fitness value, and the individuals are given the crossover probability at the same time. (2) According to the above crossover probability of individuals, two individuals are chosen randomly, and the two individuals can participate in crossover operation when the crossover conditions are meeting. (3) The binary sequence is generated randomly as intersection and the length of the binary sequence is the same as the number of parameters for individuals. (4) Crossover operation is performed on intersection for the two individuals. For more details on the crossover operation of GA, please refer to Goldberg [27].

**Figure 2:**
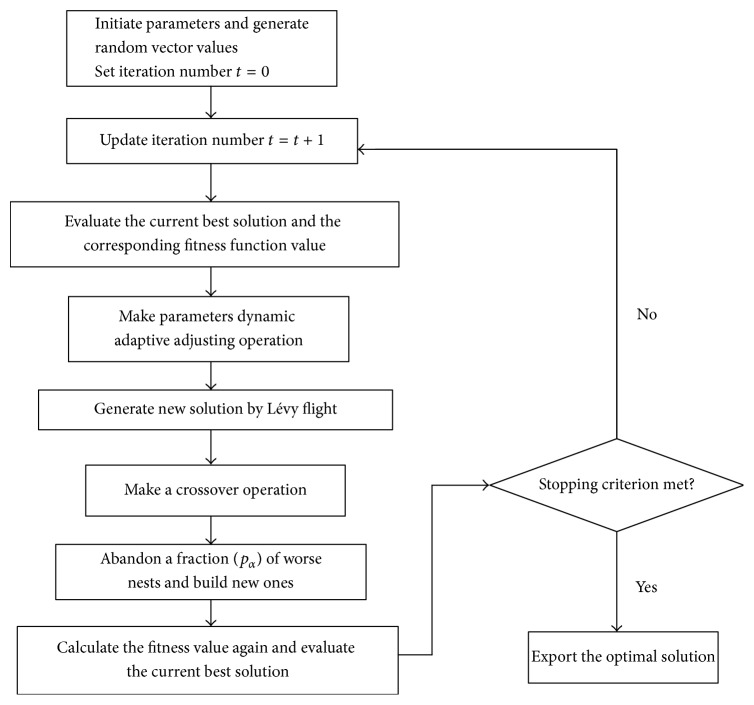
Flowchart of the DACS-CO algorithm.

The whole scheme of the DACS-CO algorithm is shown in Figure 2.

## 4. Simulation Experiment

### 4.1. Experiment Layout

**Figure 3:**
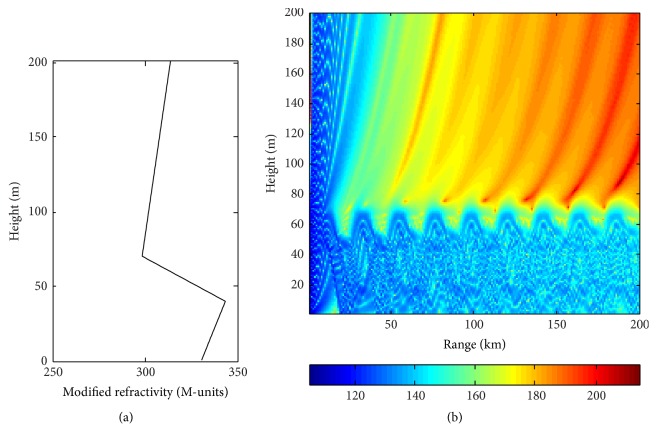
Trilinear M-profile and its corresponding propagation loss coverage diagram.

The numerical simulation experiments are designed as follows: (1) Select radar system parameters: radar frequency of 8.0 GHz, beamwidth of 1.5, antenna height of 16.0 m, and so forth. Set an M-profile with true parameter vector *m* = (0.33, −1.5,40,30). Lower and upper search limits of (0, −2,25,0) and (0.5, −0.5,50,50) are set. (2) Calculate radar electromagnetic wave propagation loss, which is simulated as the observation value. (3) Take DACS-CO algorithm to estimate atmospheric refractivity parameters.  Figure 3(a) shows the modified refractivity profile of the M-profile with true parameter.  Figure 3(b) shows the corresponding propagation loss coverage diagram calculated by the terrain parabolic equation.

### 4.2. Experiment Result

To perform RFC simulation experiments, the parameters of DACS-CO algorithm are set as follows: population size, *n* = 20; probability of abandoning the worse nests *p*_*α*_, *p*_*α* min_ = 0, *p*_*α* max_ = 1, and *p*_*α* initial_ = 0.25, *l*_*p*_*α*__ = 1.05; step size *α*, *α*_min_ = (*x*_*u*_ − *x*_*l*_)/5000, *α*_max_ = (*x*_*u*_ − *x*_*l*_)/100, and *α*_initial_ = (*x*_*u*_ − *x*_*l*_)/1000, *l*_*α*_ = 2, where *x*_*u*_ and *x*_*l*_ are the up and low boundary of the solution; the crossover rate, *P*_*c*_ = 0.5. In order to illustrate the superiority of DACS-CO, the retrieval results are compared with the CS, GA, and PSO algorithm. The parameters of those algorithms are set as follows: the maximum generation number, *N*_max_ = 100, CS (*α* = 0.01, *p*_*α*_ = 0.25), GA (*P*_*c*_ = 0.7, *P*_*m*_ = 0.01), where *P*_*c*_ is the crossover rate and *P*_*m*_ is the mutation rate, and PSO (*ω* = 0.8, *c*_1_ = *c*_2_ = 2), where *ω* is the inertia weight and *c*_1_ and *c*_2_ are the learning factors. The statistical results for atmospheric duct parameters using synthetic data are shown in Table 1.

**Figure 4:**
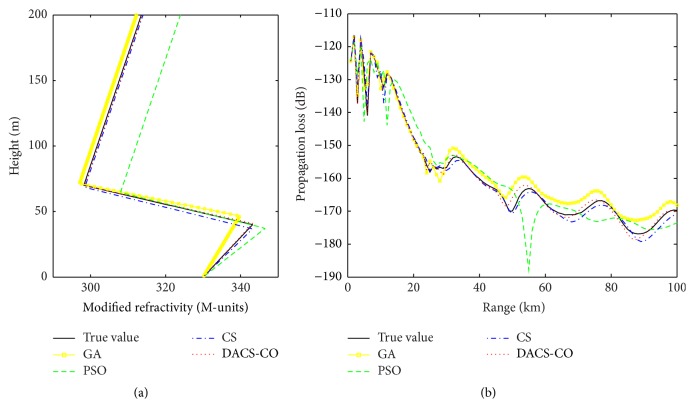
Inversion results of DACS-CO, CS, GA, and PSO for synthetic data. (a) The modified refractivity profile. (b) The corresponding propagation loss coverage diagram of different algorithms.

The smaller the relative error (RE), the better the retrieval result. Thus, it can be seen from Table 1 that the retrieval values obtained from DACS-CO algorithm are obviously more accurate than those obtained from CS, GA, and PSO algorithm. The retrieval values of DACS-CO algorithm are very close to the true values. Especially for mixed layer slope *c*_1_, the relative error (RE) is just 2.1%, which is important for precision improvement.  Figure 4 shows the M-profile inversions and the corresponding propagation loss coverage diagram based on the results in Table 1. From Figure 4, it can be seen that the M-profile and clutter plots of DACS-CO are more close to the true M-profile and propagation loss than the other three algorithms, which shows the obvious superiority of DACS-CO algorithm.

**Figure 5:**
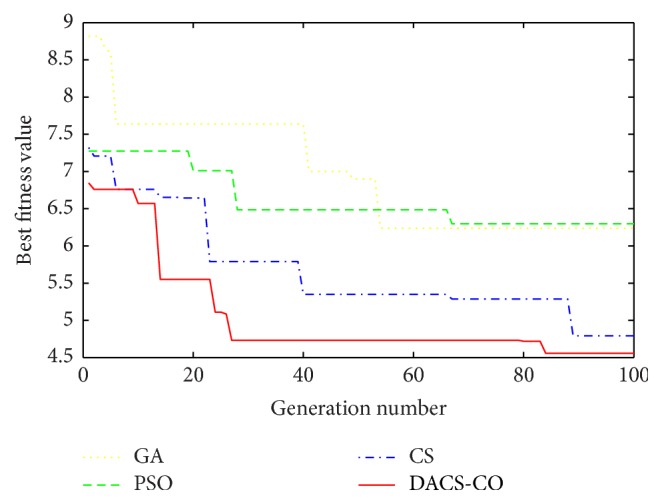
The convergence process of the best fitness value during the iterations run by different algorithms for synthetic data.

Figure 5 illustrates the convergence process of the best fitness values during the iterations. The lower the best fitness value, the better the inversion result. It can be seen obviously from Figure 5 that the best fitness values obtained from DACS-CO algorithm are quite better than those from the GA, PSO, and CS algorithms. The calculating speed of DACS-CO converged to the optimal solution is much quicker than the other algorithms. It can be concluded that the DACS-CO algorithm contributes to superior performance compared with the comparison algorithms. The DACS-CO algorithm has higher efficiency and accuracy than the other three algorithms in the retrieval of atmospheric refractivity parameters.

## 5. Real Data Experiment

**Figure 6:**
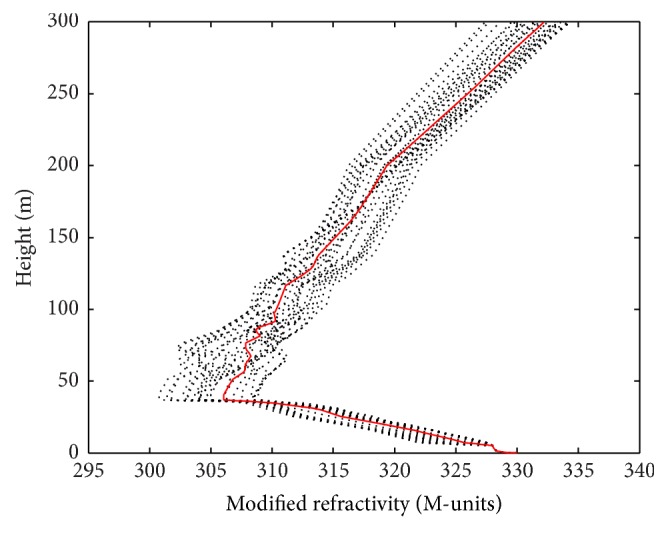
The 32 observed profiles measured from helicopter (dotted) and the average value of the 32 profiles (solid line).

In order to verify the validation of DACS-CO algorithm with real radar clutter, the observed data obtained from the Wallops Island on April 2, 1998, experiment is selected. The radar clutter data were gathered by the space range radar (SPANDAR). The refractivity profiles were obtained by an instrumented helicopter and the data include 32 profiles in the horizontal range of 60 km. For more detailed Wallops98 experiment, please refer to [1].  Figure 6 shows the observed 32 profiles measured from helicopter (dotted) and the average value of the 32 profiles (solid line). It can be seen that the error of those 32 profiles is mostly within 5 M. When the observation environment does not exhibit strong convection weather phenomenon, the environment of measured data can be approximately horizontal homogeneous environment [28]. Thus, according to the actual computing needs, we select the average value of the 32 profiles as the measured profile in this study.

**Figure 7:**
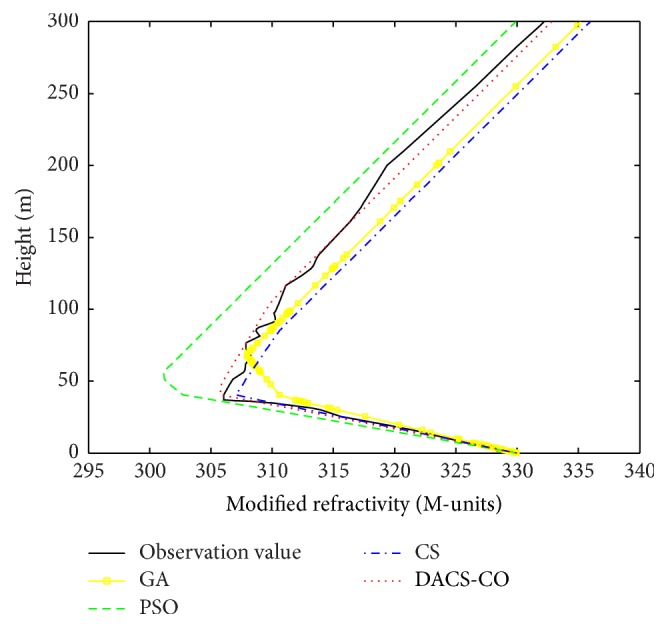
The inversion results of modified refractivity profile based on the Wallops98 data.

**Figure 8:**
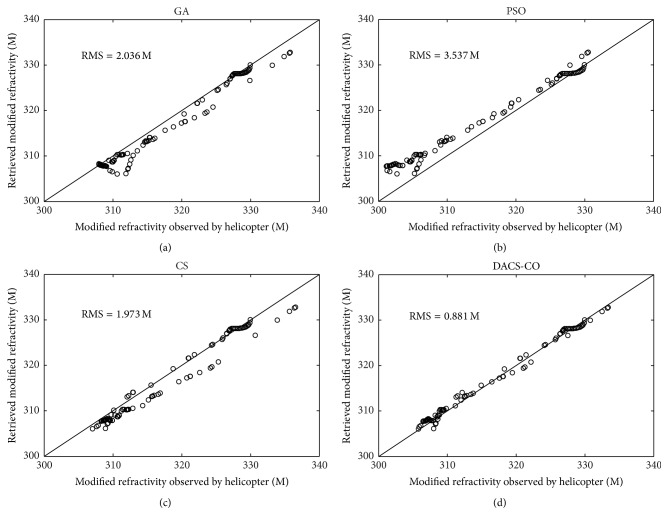
Inversion results based on the Wallops98 data. (a) Scatter plot of the retrieved modified refractivity for GA. (b) Scatter plot of the retrieved modified refractivity for PSO. (c) Scatter plot of the retrieved modified refractivity for CS. (d) Scatter plot of the retrieved modified refractivity for DACS-CO.

Here, the same four-parameter model and parameters setting of DACS-CO, CS, GA, and PSO algorithms used in the simulation experiment case are selected. The lower and upper search limits are set as (−1, −1,10,0) and (0,1, 75,75) [7]. The radar clutter data got from the SPANDAR are inverted using those four algorithms and the results were compared with the profile obtained from helicopter.  Figure 7 shows the inversion results of modified refractivity profile based on the Wallops98 data. From Figure 7, it can be seen that the modified refractivity profile obtained from DACS-CO is very close to the measured refractivity profile and more accurate than the other three algorithms.  Figure 8 shows inversion results based on the Wallops98 data.  Figure 8(a) is the scatter plot of the retrieved modified refractivity and modified refractivity obtained from helicopter for GA algorithm. It can be seen that these points are much more dispersed and most of these points deviate the isoline. The RMS is 2.036 M. Figures 8(b) and 8(c) are the scatter plot for PSO and CS. These points are also relatively scattering. The RMS is 3.537 M and 1.973 M, respectively.  Figure 8(d) is the retrieval result of DACS-CO algorithm. The points are concentrated near the isoline and the RMS is 0.881 M, which is much smaller than GA, PSO, and CS. From Figures 7 and 8, the retrieved results demonstrate that the modified refractivity profiles obtained from DACS-CO algorithm are obviously more accurate than those obtained from GA, PSO, and CS algorithms.

**Figure 9:**
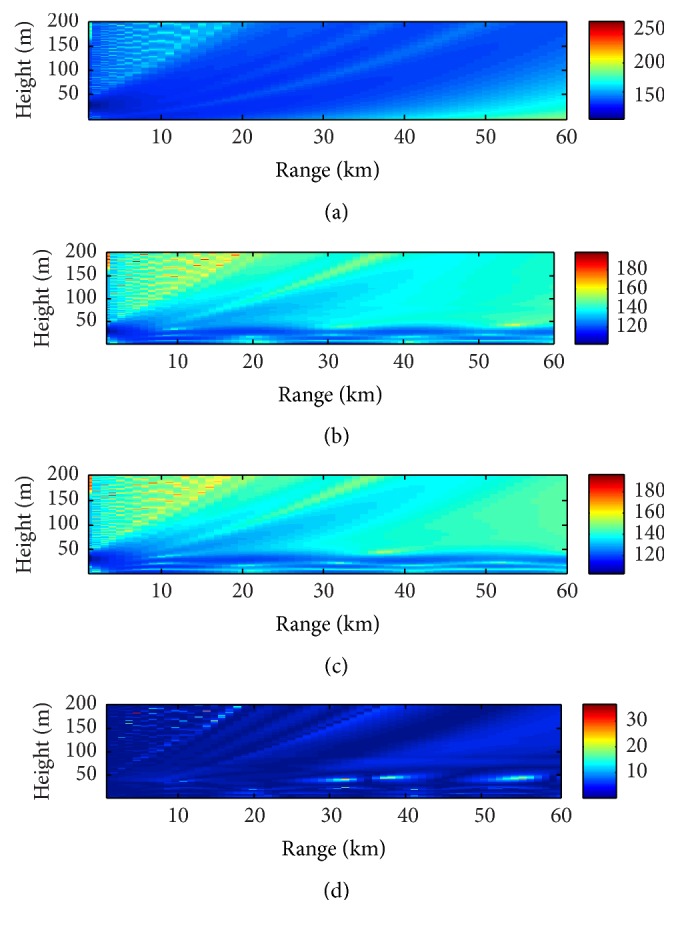
Inversion results based on the Wallops98 data. (a) Coverage diagram (dB) based on the standard atmosphere (0.118 M-units/m). (b) Coverage diagram (dB) based on the refractivity profile measured from helicopter. (c) Coverage diagram (dB) based on the inverted profile of DACS-CO algorithm. (d) Difference (dB) between coverage diagrams (b) and (c).

Using the radar clutter to estimate the atmospheric refractivity profile, the objective is not to obtain an accurate profile, but to get one profile that can well describe the basic information of the atmospheric refractivity environment.  Figure 9 summarizes the electromagnetic waves propagation information based on the Wallops98 data using the DACS-CO algorithm.  Figure 9(a) shows the coverage diagram obtained from a standard atmospheric condition. The coverage diagram in Figure 9(b) is obtained from the refractivity profile measured by helicopter.  Figure 9(c) displays the coverage diagram based on the inverted profile of DACS-CO algorithm.  Figure 9(d) is the difference between Figures 9(b) and 9(c), and the value is mostly less than 10 dB. From Figure 9, it can be seen that the atmospheric refractivity profile inverted from DACS-CO algorithm can well describe the basic information of electromagnetic waves propagation characteristics in the atmospheric duct environment.

## 6. Conclusion

In this paper, a novel hybrid metaheuristic algorithm, DACS-CO, is a technique used to estimate atmospheric refractivity in the RFC method. The hybrid algorithm integrates the parameter dynamic adaptive adjusting process and crossover operation into the standard cuckoo search. The dynamic adaptive adjusting operation of DACS-CO algorithm can improve the convergence speed and the quality of solution of CS algorithm. In addition, the crossover operation of DACS-CO can exchange information between individuals to improve population diversity. The numerical simulation experiments demonstrate that the hybrid algorithm could retrieve atmospheric refractivity parameters with more precision and efficiency than CS, GA, and PSO algorithms. And the real radar clutter experiments illustrate that the refractivity profile obtained from DACS-CO algorithm can well describe the basic information of the atmospheric refractivity environment. Thus, it can be concluded that the optimization algorithm, DACS-CO, can provide a more precise and efficient method for near-real-time estimation of atmospheric refractivity from radar clutter.

## Figures and Tables

**Table 1:** Synthetic data: the statistical results for atmospheric duct parameters estimation of DACS-CO, CS, GA, and PSO algorithm.

Parameter	True value	Retrieval value				Relative error (RE)			
		GA	PSO	CS	DACS-CO	GA	PSO	CS	DACS-CO
*c*_1_ (M-units/m)	0.33	0.21	0.446	0.359	0.323	36.40%	35.15%	8.91%	2.10%
*c*_2_ (M-units/m)	−1.5	−1.729	−1.418	−1.417	−1.573	15.30%	5.47%	5.57%	4.87%
*h*_1_ (m)	40	46.569	37.244	36.788	40.98	16.40%	6.89%	8.03%	2.40%
*h*_2_ (m)	30	24.706	27.249	31.601	28.536	17.60%	9.17%	5.34%	4.88%
